# Group A *Streptococcus* among American Indian Persons, White Mountain Apache Tribal Lands, United States, 2016–2019^1^

**DOI:** 10.3201/eid3108.240765

**Published:** 2025-08

**Authors:** Catherine G. Sutcliffe, Ryan Close, Laura B. Brown, Dennie Parker, Jayshree Patel, Eugene Romancito, Robert Weatherholtz, James McAuley, Laura L. Hammitt

**Affiliations:** Johns Hopkins Bloomberg School of Public Health, Baltimore, Maryland, USA (C.G. Sutcliffe, L.B. Brown, D. Parker, R. Weatherholtz, L.L. Hammitt); Indian Health Service Hospital, Whiteriver, Arizona, USA (R. Close, J. Patel, E. Romancito, J. McAuley); MaineHealth, Portland, Maine, USA (R. Close)

**Keywords:** Group A *Streptococcus*, streptococci, American Indian, Native American, bacteria, epidemiology, whole-genome sequencing, United States

## Abstract

American Indian populations have higher rates of invasive disease because of group A *Streptococcus* (GAS). This study describes the rates of severe and invasive GAS (siGAS) infections and the distribution of circulating *emm* types among nonsevere and siGAS cases in the White Mountain Apache Tribal lands in Arizona, USA, during 2016–2019. Isolates underwent whole-genome sequencing to determine *emm* type. Among siGAS cases, 36% of patients were female, the median age was 40.7 years, and 47.2% of patients were co-infected with *Staphylococcus aureus*. The age-standardized incidence rate during 2018–2019 was 554.2/100,000 persons. Among the pharyngitis isolates from 2017–2018, the most common *emm* types were 82 (36.3%), 6 (22.2%), and 60 (16.3%). Among the siGAS cases in 2017–2019, the most common *emm* type was 82 (65.5%) in the first year and 91 (36.2%) in the second year. Interventions are needed to address the high rates of GAS disease in this population.

*Streptococcus pyogenes* (group A *Streptococcus* [GAS]) causes multiple clinical conditions, from noninvasive pharyngitis and impetigo to more invasive streptococcal toxic shock syndrome and necrotizing fasciitis (*1*). Globally, invasive GAS (iGAS) infections are estimated to cause >160,000 deaths annually, and poststreptococcal conditions, primarily rheumatic heart disease, are estimated to cause an additional 476,000 deaths annually (*2*,*3*). In the United States, the increased severity of GAS infections and rising rates of iGAS were first described in the 1980s (*4*). After several decades of stable incidence rates, rates increased from <4.0 cases to 7.6 cases/100,000 persons in 2019 (*5*–*7*; Bact Facts Interactive Data Dashboard, https://www.cdc.gov/abcs/bact-facts/data-dashboard.html). After decreasing during the COVID-19 pandemic, rates continued to increase to 8.2 cases/100,000 persons in 2022 (Bact Facts Interactive Data Dashboard. 

American Indian populations have disproportionately high rates of infectious disease related death compared with the general US population (*8*,*9*). However, American Indian populations are underrepresented in national surveillance systems for iGAS, such as the Active Bacterial Core (ABC) surveillance program of the Centers for Disease Control and Prevention. This underrepresentation limits understanding of the epidemiology and rates of iGAS in the United States. A more complete understanding is critical to inform disease prevention strategies, including the development of vaccines against GAS (*10**,**11*)*.*

Studies from the 1980s in Arizona and New Mexico suggested rates of iGAS among American Indian persons are 8–10 times higher than for other ethnic groups (*4*,*12*). A more recent study in Alaska during 2001–2013 found Alaska Native persons accounted for nearly half of Alaska’s reported iGAS cases and had a rate >3 times higher than non–Alaska Native persons, despite comprising only 20% of the state’s population (*13*).

In the fall of 2016, the Indian Health Service (IHS) hospital in Whiteriver, Arizona, USA, which serves the White Mountain Apache (WMA) community, experienced several GAS-related hospitalizations, including 2 invasive cases. To better understand the rate of disease and epidemiology of iGAS in this vulnerable community, we conducted a series of studies, including active laboratory-based surveillance of all hospitalizations secondary to GAS infection. The objectives of this analysis were to describe the rates of severe and invasive GAS infections, the clinical manifestation and underlying medical conditions of severe and invasive cases, and the distribution of circulating M protein gene (*emm*) types among nonsevere, severe, and invasive cases during 2016–2019.

## Methods

### Study Setting and Overview

We conducted study activities during August 2016–February 2019 in the WMA Tribal lands, which cover an area of 2600 square miles (*2*) in eastern Arizona and have a population of ≈17,000 Tribal members. The population is served by 1 main IHS facility that provides inpatient and outpatient care and a smaller IHS outpatient clinic. A private health facility, with inpatient and outpatient services, also serves Tribal members and is located 15 miles from the WMA Tribal lands.

We undertook 3 activities in response to an apparent increase in GAS cases at the IHS hospital. First, we collected samples from 19 GAS positive cultures during August–October 2016, irrespective of infection site, clinical syndrome, or severity. We collected a limited set of demographic and clinical information and sequenced the isolates to determine *emm* types. Second, we performed active, laboratory-based surveillance for GAS pharyngitis during May 2017–August 2018, including all patients with a clinical manifestation consistent with pharyngitis who had 2 swabs collected for testing (1 for rapid antigen testing and 1 for traditional culture in the event the rapid antigen test was negative). We monitored the cultures for GAS and collected positive isolates. We collected the patient’s age and sequenced the isolates to determine *emm* types. Finally, we conducted active, laboratory-based surveillance for severe and invasive GAS infections during March 2017–February 2019. We included all cases that met eligibility criteria and conducted a chart review. We collected and sequenced isolates to determine *emm* types.

### Active, Laboratory-Based Surveillance for Invasive and Severe GAS

We conducted active, laboratory-based surveillance for severe and invasive GAS infections over a 2-year period; year 1 was March 1, 2017–February 28, 2018, and year 2 was March 1, 2018–February 28, 2019. At the IHS hospital, patient specimens were collected at the discretion of the clinical provider; we then sent isolates to the Johns Hopkins Center for Indigenous Health (CIH) laboratory in Whiteriver for processing and storage. We initiated surveillance at the private facility in August 2018. We retrospectively identified all cases of severe and invasive GAS infection that occurred during March 1, 2017–July 31, 2018, through review of microbiology reports and medical charts. During August 1, 2018–February 28, 2019, we identified cases prospectively and sent isolates to the CIH laboratory. At both facilities, we obtained information on case demographics (age, sex, race), underlying medical conditions, clinical syndrome (on the basis of physician report), co-infections (identified on the hospital laboratory report), and health outcomes (amputation or death within 30 days of the initial culture) by using chart review.

We defined a case of iGAS as an American Indian patient living in a community in or near the WMA Tribal lands who had GAS isolated from a normally sterile body site (e.g., blood, cerebrospinal fluid) or from a wound with a diagnosis of streptococcal toxic shock syndrome or necrotizing soft tissue infection, including necrotizing fasciitis. We defined a case of severe GAS infection as an American Indian patient living in a community in or near the WMA Tribal lands who had GAS isolated from a nonsterile site (e.g., wound, ear) and who required hospitalization that otherwise did not meet invasive criteria. We considered patients with multiple isolates collected within 7 days of the initial culture the same case. We defined a reoccurring case as a patient with a new case event, which was GAS isolated from specimens collected >8 days after the initial date of culture.

### Laboratory Methods

At the IHS laboratory, positive blood cultures were identified by using the BACTEC FX automated blood culture system (Becton, Dickinson and Company, https://www.bd.com) for samples collected in BACTEC (Becton, Dickinson and Company) aerobic and anaerobic blood culture bottles. When growth was detected, the positive blood cultures were subcultured to MacConkey, chocolate, and sheep’s blood agar plates. Wound and throat samples were collected by using liquid double swabs (Becton Dickinson) and plated onto sheep’s blood agar with a bacitracin disc.

At the private facility, positive blood cultures were identified by using the VITEK 2 automated blood culture system (bioMérieux, https://www.biomerieux.com). When growth was detected, GAS was confirmed by using a latex agglutination test.

As part of the surveillance for invasive and severe GAS infections, we subcultured eligible isolates in the CIH laboratory on sheep’s blood agar plates with a bacitracin disc to confirm the presence of GAS. After 24 hours of incubation, we stored the cultured colonies in skim milk at −80°C.

We sent all the recovered isolates to the Musser laboratory at Houston Methodist Research Institute (Houston, TX, USA) for whole-genome sequencing to determine *emm* types. Strain growth, isolation of chromosomal DNA, generation of paired-end libraries, and multiplexed sequencing by using an Illumina NextSeq 550 (Illumina, https://www.illumina.com) were performed as previously described (*14*–*16*). Reads were preprocessed by using Trimmomatic and Musket (*17*,*18*) and then assembled de novo by using SPAdes (*19*). Gene content data were generated by using short-read sequence typer 2 (*20*) and custom databases as previously described (*21*).

### Statistical Analysis

We compared the characteristics and outcomes of invasive and severe cases from year 1 and year 2 by using χ^2^ or Fisher exact tests for categorical variables and Wilcoxon rank-sum tests for continuous variables. We calculated incidence rates of invasive and severe GAS infections (overall and separately) by using the IHS user population during 2017–2018 as the denominator for years 1 and 2. IHS defines users as any American Indian patient receiving services at the IHS facility in the preceding 3 years (*22*). We included all cases identified from the surveillance system from communities included in the IHS user population for the IHS facility in the numerator, regardless of whether an isolate was collected. For each year and type of infection, we calculated incidence overall and by age by using Poisson regression with robust variance estimation to account for recurrent infections. For comparison with the general US population (Bact Facts Interactive Data Dashboard), we calculated age-standardized incidence rates for each year by using direct standardization methods by using US census data from 2017 as the reference (*23*).

We summarized the distribution of *emm* types by sample type (clinical, pharyngitis, severe or invasive isolates), year, and patient characteristics (age, sex, clinical manifestation). We separately estimated the proportion of *emm* types targeted by an experimental 30-valent type-specific vaccine for pharyngitis and severe or invasive isolates (*24*,*25*). We conducted analyses by using SAS software version 9.4 (SAS Institute, https://www.sas.com) and Stata software version 14.2 (StataCorp, LLC, https://www.stata.com).

### Ethics Statement

This study was approved by the WMA Tribe and by the Institutional Review Boards of the Phoenix Area IHS (approval no. PXR 18.06) and the Johns Hopkins Bloomberg School of Public Health (approval no. 8510). A Health Insurance Portability and Accountability Act waiver was obtained to conduct medical chart reviews.

## Results

### Effect of Severe and Invasive GAS

During the surveillance period, 48 invasive cases (23 cases in year 1 and 25 in year 2) and 113 severe cases (56 cases in year 1 and 57 in year 2) were detected. The 161 cases occurred among 146 persons: 9 persons had 1 recurrent infection, and 3 persons had 2 recurrent infections.

Among the 48 invasive cases, 52 isolates were identified: 32 (61.5%) from blood, 1 (1.9%) from synovial fluid, and 19 (36.5%) from wounds. Among the 113 severe cases, 113 isolates were identified: 106 (93.8%) from wounds, 5 (4.2%) from abscesses, 1 (0.9%) from a peritonsillar abscess, and 1 (0.9%) from an ear culture in a patient with mastoiditis.

Among the GAS cases (Table 1), compared with those with severe cases, patients with invasive cases were older, were more likely to have underlying conditions of hypertension and heart failure, were more likely to have pneumonia and sepsis, and had much longer hospitalization lengths. Patients with severe cases were much more likely to have alcoholism as an underlying condition and to have cellulitis as a disease syndrome. The clinical syndromes associated with severe and invasive infections differed by age: skin infections (e.g., eczema, impetigo) were dominant among children <5 years of age; trauma (e.g., falls and self-inflicted wounds) and skin infections (e.g., insect bites, sores, blisters) were dominant among older children and adolescents; trauma (e.g., lacerations, burns, injuries) was dominant among adults 18–49 years of age; and trauma (e.g., burns and falls) and complications of underlying conditions (e.g., prior amputation or diabetic foot ulcers) were dominant among older adults (Appendix 1 Table 1).

**Table 1:** Demographic and clinical characteristics of invasive and severe group A *Streptococcus* infections among American Indian persons in the White Mountain Apache Tribal lands, Arizona, USA, 2017–2019*

Characteristic	Total, n = 161†	Invasive, n = 48	Severe, n = 113	p value‡
Sex				0.33
F	58 (36.0)	20 (41.7)	38 (33.6)	
M	103 (64.0)	28 (58.3)	75 (66.4)	
Median age, y (IQR)	40.7 (30.5–55.8)	51.3 (39.3–65.4)	39.4 (28.6–49.6)	<0.001
Age, y				0.01
<1	4 (2.5)	1 (2.1)	3 (2.7)	
1–4	3 (1.9)	0	3 (2.7)	
5–17	11 (6.8)	0	11 (9.7)	
18–34	36 (22.4)	6 (12.5)	30 (26.6)	
35–49	54 (33.5)	16 (33.3)	38 (33.6)	
50–64	32 (19.9)	12 (25.0)	20 (17.7)	
65–74	12 (7.5)	8 (16.7)	4 (3.5)	
>75	9 (5.6)	5 (10.4)	4 (3.5)	
Residence before culture				0.43
Private residence	151 (93.8)	45 (93.8)	106 (93.8)	
Long-term care facility	2 (1.2)	1 (2.1)	1 (0.9)	
Unhoused	5 (3.1)	1 (2.1)	4 (3.5)	
Incarcerated	1 (0.6)	1 (2.1)	0	
Unknown	2 (1.2)	0	2 (1.8)	
Body mass index§				0.18
<25	33 (23.4)	9 (20.0)	24 (25.0)	
25–29	39 (26.7)	9 (20.0)	30 (31.3)	
>30	69 (48.9)	27 (60.0)	42 (43.8)	
Underlying conditions¶				
Alcoholism	90 (55.9)	20 (41.7)	70 (62.0)	0.02
Hypertension	61 (37.9)	25 (52.1)	36 (31.9)	0.02
Diabetes	54 (33.5)	21 (43.8)	33 (29.2)	0.07
Chronic skin breakdown	33 (20.5)	8 (16.7)	25 (22.1)	0.43
Smoker	16 (9.9)	2 (4.2)	14 (12.4)	0.15
Cirrhosis	14 (8.7)	4 (8.3)	10 (8.9)	1.00
Asthma	14 (8.7)	4 (8.3)	10 (8.9)	1.00
Renal failure/dialysis	14 (8.7)	6 (12.5)	8 (7.1)	0.26
Peripheral vascular disease	12 (7.5)	5 (10.4)	7 (6.3)	0.36
Abscess	12 (7.5)	4 (8.3)	8 (7.1)	0.75
Heart failure	11 (6.8)	7 (14.6)	4 (3.5)	0.02
Immunosuppression	8 (5.0)	1 (2.1)	7 (6.2)	0.44
Atherosclerosis	6 (3.7)	1 (2.1)	5 (4.4)	0.67
Transplant	5 (3.1)	1 (2.1)	4 (3.5)	0.63
Chronic obstructive pulmonary disease	4 (2.5)	2 (4.2)	2 (1.8)	0.58
Cognitive deficit	4 (2.5)	2 (4.2)	2 (1.8)	0.58
Myocardial infarction	4 (2.5)	1 (2.1)	3 (2.7)	1.00
Dementia	3 (1.9)	1 (2.1)	2 (1.8)	1.00
Burns	3 (1.9)	0	3 (2.7)	0.56
Hemiplegia	2 (1.2)	0	2 (1.8)	1.00
Connective tissue disorder	2 (1.2)	0	2 (1.8)	1.00
Stroke	2 (1.2)	2 (4.2)	0	0.09
Malignancy	1 (0.6)	1 (2.1)	0	0.30
Peptic ulcer	1 (0.6)	0	1 (0.9)	1.00
HIV	0	0	0	NA
Injection drug use	0	0	0	NA
Any	161 (100.0)	48 (100.0)	113 (100.0)	NA
Severe GAS infection within previous 5 y#	23 (14.6)	9 (19.2)	14 (12.6)	0.45
Disease syndromes associated with GAS infection¶				
Pneumonia	6 (3.7)	5 (10.4)	1 (0.9)	0.009
Meningitis	2 (1.2)	1 (2.1)	1 (0.9)	0.51
Cellulitis	142 (88.2)	34 (70.8)	108 (95.5)	<0.0001
Osteomyelitis	17 (10.6)	5 (10.4)	12 (10.6)	0.97
Sepsis	41 (25.5)	24 (50.0)	17 (15.0)	<0.0001
Arthritis	4 (2.5)	1 (2.1)	3 (2.7)	1.00
Endometritis**	1 (1.7)	1 (5.0)	0	0.34
Otitis media	1 (0.6)	0	1 (0.9)	1.00
Streptococcal toxic shock syndrome	8 (5.0)	8 (16.7)	0	<0.0001
Necrotizing soft tissue infection	14 (8.7)	14 (29.2)	0	<0.0001
Necrotizing fasciitis	11 (6.8)	11 (22.9)	0	<0.0001
Recent triggers within previous 14 d¶				
Surgery or skin incision	5 (3.1)	1 (2.1)	4 (3.5)	1.00
Delivery**	1 (1.7)	1 (5.0)	0	0.34
Postpartum**	1 (1.7)	1 (2.6)	0	0.27
Penetrating trauma	51 (31.7)	12 (25.0)	39 (34.5)	0.23
Blunt force trauma	23 (14.3)	7 (14.6)	16 (14.2)	0.94
Surgical wound (postoperative)	3 (1.9)	2 (4.2)	1 (0.9)	0.21
Burns	3 (1.9)	1 (2.1)	2 (1.8)	1.00
None	84 (52.2)	26 (54.2)	58 (51.3)	0.74
Unknown	4 (2.5)	0	4 (3.5)	0.39
Admitted to the hospital††	157 (97.5)	45 (93.8)	112 (99.1)	0.05
Median length of hospitalization, d (IQR, total range)	4 (3–7, 0–27)	5.5 (4–12, 2–27)	4 (3–6, 0–24)	0.004

*Values are no. (%) except as indicated. GAS, group A *Streptococcus*; IQR, interquartile range; NA, not applicable. †Twenty-five cases were identified from the private medical facility, including 22 through retrospective review for which isolates were not collected. ‡p value comparing invasive and severe cases from χ^2^ test or Fisher exact test for categorical variables and Wilcox rank sum tests for continuous variables. §Among patients >18 years of age; missing for 2 persons. ¶Multiple conditions or syndromes or triggers per case. #Includes 4 recurrent cases during the study period. **Among women only (n = 58); postpartum defined as <30 days postdelivery. ††One severe case was offered admission but refused.

Almost half of patients were co-infected with *Staphylococcus aureus* (47.2%). Those with severe cases were significantly more likely to be co-infected with *S. aureus* than were those with invasive cases (59.3% vs. 18.8%; p<0.0001). Among patients co-infected with *S. aureus*, 44.7% were co-infected with methicillin-resistant *S. aureus*, at similar rates for severe (44.8%) and invasive (44.4%; p = 0.99) cases. Similar results were found when restricting the data to cases with GAS isolated from a wound: those with severe cases (61.3%) were more likely to be co-infected than were those with invasive cases (35.0%), and a similar proportion of co-infections were methicillin-resistant *S. aureus* (45.8%; severe, 46.2%; invasive, 42.9%; p = 0.87).

Overall, 6 (3.7%) patients had an amputation because of the GAS infection, 3 (6.7%) of those with invasive cases and 3 (2.6%) of those with severe cases (p = 0.22). Three (1.9%) patients died within 30 days of the initial culture; all had invasive infections (6.3%; p = 0.002).

Antimicrobial resistance testing at the clinical laboratories was only performed on the 32 invasive isolates identified from blood. No resistance was identified to the cephalosporins, penicillins, or fluoroquinolones tested (Appendix 1 Table 2). Only 6 (18.8%) isolates demonstrated resistance; all were resistant to tetracycline, and 3 (9.4%) were also resistant to clindamycin.

The overall incidence of severe and invasive GAS infections during the surveillance period was 472.7 (95% confidence interval [CI] 405.2–551.4)/100,000 persons (Table 2). The incidence of invasive infections was 140.9 (95% CI 106.2–187.0)/100,000 persons and the incidence of severe infections was 331.8 (95% CI 276.0–398.8)/100,000 persons. Rates did not vary significantly by year (Table 3). For both severe and invasive infections, rates were higher for adults than children; the highest rates of severe infections were observed among adults 18–49 years of age and invasive infections were observed among adults >65 years of age. The overall age-standardized incidence of severe and invasive infections was 554.2/100,000 persons. Separately, the age-standardized incidence of severe infections was 359.6/100,000 persons and of invasive infections was 194.6/100,000 persons.

**Table 2:** Incidence rates of severe and invasive group A *Streptococcus* infection among American Indian persons in the White Mountain Apache Tribal lands, Arizona, USA, 2017–2019

Characteristic	Total no.	Severe and invasive infections			Invasive infections			Severe infections	
		No.	Incidence* (95% CI)		No.	Incidence* (95% CI)		No.	Incidence* (95% CI)
Overall	34,061	161	472.7 (405.2–551.4)		48	140.9 (106.2–187.0)		113	331.8 (276.0–398.8)
By age, y									
0–4	3,391	7	206.4 (98.5–432.7)		1	29.5 (4.2–209.4)		6	176.9 (79.5–393.6)
5–17	8,791	11	125.1 (69.3–225.9)		0	0		11	125.1 (69.3–225.9)
18–49	14,514	90	620.1 (504.7–761.9)		22	151.6 (99.8–230.1)		68	468.5 (369.6–593.9)
50–64	4,845	32	660.5 (467.6–932.9)		12	247.7 (140.8–435.9)		20	412.8 (266.6–639.3)
>65	2,520	21	833.3 (544.3–1,275.8)		13	516.0 (300.0–887.3)		8	317.5 (158.9–634.1)
Year 1†	16,948	79	466.1 (374.1–580.8)		23	135.7 (90.2–204.2)		56	330.4 (254.4–429.2)
By age, y									
0–4	1,693	5	295.3 (123.1–708.8)		1	59.1 (8.3–419.3)		4	236.3 (88.8–629.0)
5–17	4,366	5	114.5 (47.7–275.0)		0	0		5	114.5 (47.7–275.0)
18–49	7,260	42	578.5 (427.9–782.1)		8	110.2 (55.1–220.3)		34	468.3 (334.9–654.9)
50–64	2,383	14	587.5 (348.5–990.5)		6	251.9 (113.3–560.0)		8	335.7 (168.1–670.5)
>65	1,246	13	1043.3 (607.5–1,791.8)		8	642.1 (321.8–1,281.0)		5	401.3 (167.3–962.4)
Year 2†	17,113	82	479.2 (386.1–594.7)		25	146.1 (98.7–216.1)		57	333.1 (257.0–431.6)
By age, y									
0–4	1,698	2	117.8 (29.5–470.8)		0	0		2	117.8 (29.5–470.8)
5–17	4,425	6	135.6 (61.0–301.7)		0	0		6	135.6 (61.0–301.7)
18–49	7,254	48	66.2 (499.1–877.2)		14	193.0 (114.4–325.7)		34	468.7 (335.2–655.5)
50–64	2,462	18	731.1 (461.4–1,158.5)		6	243.7 (109.5–541.9)		12	487.4 (277.2–857.1)
>65	1,274	8	627.9 (314.7–1,252.9)		5	392.4 (163.6–941.3)		3	235.5 (76.1–729.2)

*Cases/100,000 persons. †No significant difference was observed between years 1 (March 2017–February 2018) and 2 (March 2018–February 2019) for all group A *Streptococcus* (incidence rate ratio: 1.03; 95% confidence interval: 0.76–1.40), invasive group A *Streptococcus* (incidence rate ratio: 1.08; 95% confidence interval: 0.61–1.90) or severe group A *Streptococcus* (incidence rate ratio: 1.01; 95% confidence interval: 0.70–1.46).

### Molecular Characteristics of GAS Isolates

**Figure 1:**
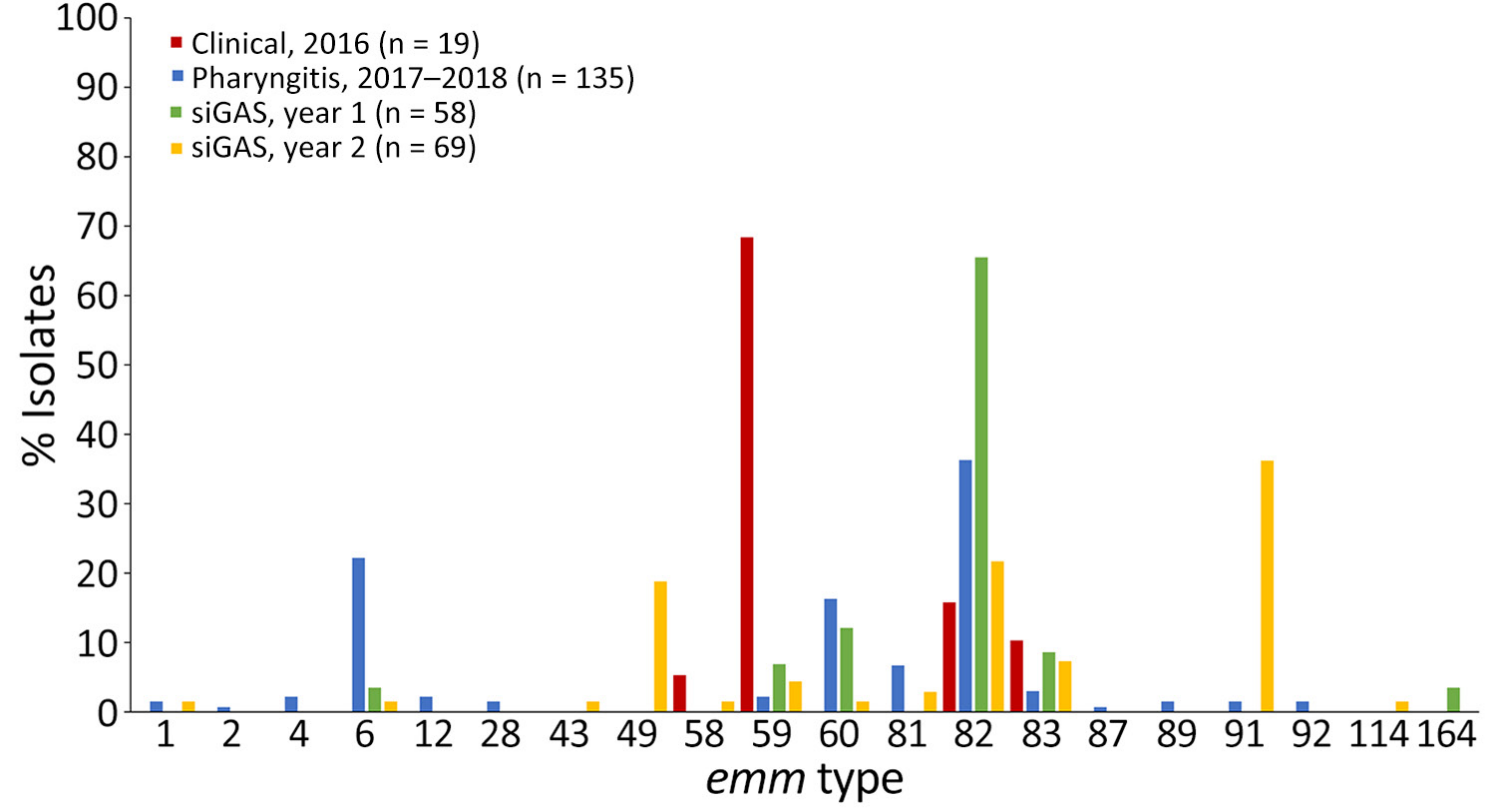
Distribution of *emm* types among cases of group A *Streptococcus* from American Indian persons in the White Mountain Apache Tribal Lands, Arizona, USA, 2016–2019. Clinical isolates were convenience samples; all other samples were collected as part of active, laboratory-based surveillance. Year 1 indicates active surveillance for siGAS during March 1, 2017–February 28, 2018, and year 2 indicates active surveillance for siGAS during March 1, 2018–February 28, 2019. siGAS, severe or invasive cases of group A *Streptococcus*.

**Figure 2:**
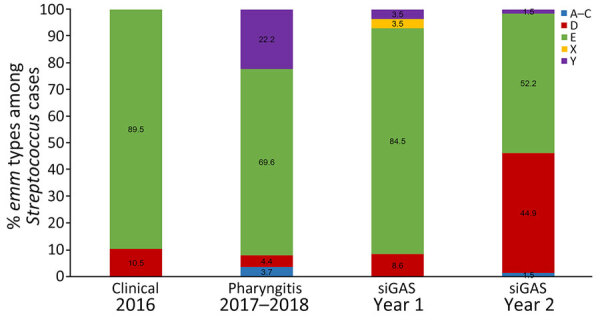
Distribution of *emm* type clusters by sample type among cases of group A *Streptococcus* from American Indian persons in the White Mountain Apache Tribal Lands, Arizona, USA, 2016–2019. Clinical isolates were convenience samples; all other samples were collected as part of active, laboratory-based surveillance. Year 1 indicates active surveillance for siGAS during March 1, 2017–February 28, 2018, and year 2 indicates active surveillance for siGAS during March 1, 2018–February 28, 2019. siGAS, severe or invasive cases of group A *Streptococcus.*

Whole-genome sequencing was completed on all 19 clinical isolates from 2016, as well as 135 of 149 pharyngitis isolates and 131 of 165 severe and invasive GAS isolates (Appendix 1 Tables 3–7; Appendix 2 Table). Four isolates were from a second source from the same invasive case and yielded the same *emm* type and were excluded from the analysis. The distribution of *emm* types varied by year and sample type (Appendix 1 Table 4; Figures 1, 2). In 2016, out of 19 clinical isolates collected, 13 (68%) were *emm*59. Among 135 pharyngitis isolates collected during 2017–2018, the most common *emm* types were 82, 60, and 6, with little variation by year (Appendix 1 Figure 2); 80% were *emm* types targeted by an experimental 30-valent type-specific vaccine (*24*). In year 1 (2017–2018) of active surveillance, *emm*82 caused most (65.5%) severe and invasive GAS infections. In year 2 (2018–2019), more *emm* types were present; 91 (36.2%), 82 (21.7%), and 49 (18.8%) were the most common. Of the 127 severe and invasive GAS isolates in this study, 66% were *emm* types targeted by the 30-valent type-specific vaccine (*24*).

## Discussion

This study, conducted in an American Indian community in Arizona during 2016–2019, revealed high rates of severe and invasive GAS infections, particularly among older adults. Co-infection with *S. aureus* was common among persons with severe skin and soft tissue infections. Although most outcomes were favorable, a small proportion of infections resulted in amputation or death. Molecular characterization of isolates found a shift in dominant *emm* types over time with overlapping distributions between pharyngitis and severe and invasive isolates.

By using a population-based, laboratory-based surveillance system, we documented a rate of iGAS of 194.6/100,000 persons for the WMA community, which was >25 times that found in the general US population and among the highest reported in the world. In 2019, the rate of iGAS in the United States was 7.6/100,000 persons (Bact Facts Interactive Data Dashboard). Similar to our study, that study found the highest rates were observed among older adults (7.5/100,000 persons among adults 35–49 of age, 10.6/100,000 persons among adults 50–64 of age, and 16.1/100,000 persons among adults >65 years of age) (Bact Facts Interactive Data Dashboard). The rate in our study was also substantially higher than that reported among American Indian persons throughout Arizona in 2017 (21.6/100,000 persons) (*26*) and among Alaska Native persons during 2001–2013 (13.7/100,000 persons) (*13*). Globally, indigenous populations are found to have disproportionately high rates of iGAS, including those in Australia (23.8–82.5/100,000 persons) (*27*–*29*), New Zealand (20.4/100,000 persons) (*30*), Fiji (17.8/100,000 persons) (*31*), and Canada (10.0–52.2/100,000 persons) (*32*).

Host characteristics, host–pathogen dynamics, and pathogen virulence all likely contribute to the disparate rates of iGAS among American Indian communities, but the proportion of disease attributable to each factor is poorly understood. Many host characteristics associated with GAS infections in North America are driven by socioeconomic differences. In Canada and the United States, outbreaks of iGAS have been associated with substance use and homelessness (*33*–*36*). Whereas homelessness was uncommon in this study, alcohol misuse was common, reported in more than half the cases. Alcohol misuse was more common among persons with severe GAS infections, which were documented predominantly among younger men. All patients reported >1 underlying condition, and diabetes and hypertension, known risk factors for iGAS (*37*,*38*), were reported in approximately one third of cases. In addition, poor household conditions, household crowding, and exposure to children with sore throats have been found to be associated with iGAS in other studies (*37*). Although those factors were not directly assessed in this study, 15% of patients had a severe GAS infection in the previous 5 years, including several persons with recurrent infections during the 2-year study period, indicating relatively common and repeated exposure to GAS in the household or community. In addition, the large proportion of *emm* types belonging to the D cluster (skin specialists) and small proportion belonging to the A–C cluster (throat specialists) among severe and invasive cases (*39*) suggest that skin and soft tissue infections, which are affected by household conditions and crowding, are a major driver of serious GAS disease. In the WMA community, families tend to be larger (4.2 vs. 3.1 in the general US population) and multigenerational (22.2% vs. 4.9% in Arizona), and a higher proportion live below the federal poverty line (40.4% vs. 12.6% in the United States) (*40*,*41*). Additional studies are needed of both GAS disease and carriage to further understand the contribution of those factors in this community.

There is an increased interest in the role of virulent *emm* types among vulnerable populations in recent years. Canada experienced an outbreak of iGAS driven by the hypervirulent *emm*59 clone beginning in 2006 (*33*), and First Nations persons were disproportionately represented among cases (*42*). Later studies revealed that clone migrated to the United States, mutated, and caused outbreaks in Wyoming, Montana, and Oregon (*43*,*44*). In the ABCs program, *emm*59 was reported almost exclusively from New Mexico in 2015, with a few isolates also identified from Oregon (*45*). In 2015, an outbreak of iGAS occurred in northern Arizona, and most cases were in American Indian persons. Most isolates (62%) were *emm*59 and genetically related to the Canada strain (*46*). Of interest, *emm*59 was the dominant type found in the clinical isolates in our study in 2016. However, the dominant type shifted to *emm*82 during 2017–2018 among both GAS pharyngitis and invasive cases and then to *emm*91 in 2018 and 2019 among invasive cases, potentially suggesting introduction and rapid circulation of different types into the community. Although other studies, particularly from Canada, have found rapid shifts in dominant types in indigenous populations (*32*), many report a variety of types with none clearly dominant (*13*,*27*,*28*,*30*,*47*). Of note, the common *emm* types identified from severe and invasive cases in this study (e.g., 49, 59, 60, 82) overlapped with those commonly identified from disadvantaged communities (e.g., persons experiencing homelessness or who inject drugs) in the ABCs program during the same period (2015–2018) (*48*). Persons experiencing homelessness or who inject drugs were also found to have higher rates of disease (≈14–80-fold higher) and were more likely to have acute skin breakdown than persons without those risks (*36*), highlighting the shared social drivers of health with indigenous communities and the potential for shared learnings from further research in these communities.

The high rates of disease observed in the WMA Tribal lands and other indigenous and vulnerable communities illustrates the need for effective interventions to decrease illness and death and address health differences. Eight GAS vaccine candidates are in development; the furthest along is an M protein–based vaccine candidate targeting 30 *emm* types that was found to be immunogenic and well tolerated in a phase 1 clinical trial (*24*,*49*). In this study, 66% of severe and invasive *emm* types and 80% of pharyngitis *emm* types would have been targeted by the vaccine, similar to the 53% coverage reported for invasive cases among First Nations populations in Alberta, Canada, during 2003–2017 (*32*). That study also observed a difference in coverage between First Nations and non–First Nations populations, with a higher coverage of 77% for the non–First Nations population (*32*). The authors also observed a major difference in *emm* cluster types between First Nations and non–First Nations populations, with a larger proportion in cluster D and smaller proportion in cluster A–C among First Nations cases (*32*). Although our study did not include a nonindigenous comparison population, the estimated coverage with the 30-valent vaccine was lower than that reported for invasive cases included in the ABCs program in 2015 (88%) in the United States (*45*). Few samples from ABCs were identified with *emm* types belonging to cluster D, and those are not well represented among *emm* types included in the vaccine (*24*,*45*). The larger proportion of *emm* types from cluster D identified from indigenous cases may explain the lower *emm* type coverage observed for Indigenous populations and between cases of severe and invasive disease and pharyngitis. Potential cross-reactivity with nonvaccine *emm* types could decrease differences between populations and increase the effectiveness of the vaccine (*24*,*25*,*50*).

In conclusion, we found high rates of severe and invasive GAS disease in this American Indian community in Arizona, USA, highlighting the need to increase representation of reservation-based American Indian populations in current laboratory and genomic surveillance systems. Vaccines to prevent GAS disease are under development but are still years from licensure. Until then, interventions that are culturally informed and promote early recognition and treatment are needed to reduce the illness and death associated with GAS infections.

Appendix 1 Additional information about group A *Streptococcus* among American Indian persons, White Mountain Apache Tribal Lands, United States, 2016–2019.

Appendix 2 Additional information about BioProject and sample information of group A *Streptococcus* isolates among American Indian persons, White Mountain Apache Tribal Lands, United States, 2016–2019.
