# Supplementary material for: Group A Streptococcus among American Indian Persons, White Mountain Apache Tribal Lands, United States, 2016–2019
Source: Emerg Infect Dis. 2025 Aug;31(8):1580–8. doi: 10.3201/eid3108.240765 (PMC12309752; doi:10.3201/eid3108.240765)
Supplement: Appendix 1 — Additional information about group A Streptococcus among American Indian persons, White Mountain Apache Tribal Lands, United States, 2016–2019. [file 24-0765-Techapp-s1.pdf]

Article DOI: https://doi.org/10.3201/eid3108.240765

EID cannot ensure accessibility for supplementary materials supplied by authors. Readers who have difficulty accessing supplementary content should contact the authors for assistance.

Group A *Streptococcus* among American Indian Persons, White Mountain Apache Tribal Lands, 2016–2019

Appendix 1

Appendix Table 1. Underlying conditions, disease syndromes, and recent triggers among cases of severe and invasive group A *Streptococcus* infection by age group, 2017–2019

Characteristics	Age group 0–4 years (n = 7)	5–17 years (n = 11)	18–49 years (n = 90)	>50 years (n = 53)
Underlying conditions*, n (%)				
Alcoholism	0	2 (18.2)	73 (81.1)	15 (28.3)
Hypertension	0	2 (18.2)	21 (23.3)	38 (71.7)
Diabetes	0	0	23 (25.6)	31 (58.5)
Chronic skin breakdown	3 (42.9)	2 (18.2)	16 (17.8)	12 (22.6)
Smoker	0	0	14 (15.6)	2 (3.8)
Cirrhosis	0	0	9 (10.0)	5 (9.4)
Asthma	0	1 (9.1)	8 (8.9)	5 (9.4)
Renal failure/dialysis	0	0	4 (4.4)	10 (18.9)
Peripheral vascular disease	0	0	1 (1.1)	11 (20.8)
Abscess	0	1 (9.1)	9 (10.0)	2 (3.8)
Heart failure	0	0	4 (4.4)	7 (13.2)
Immunosuppression	0	0	2 (2.2)	6 (11.3)
Atherosclerosis	0	0	1 (1.1)	5 (9.4)
Transplant	0	0	0	5 (9.4)
Chronic obstructive pulmonary disease	0	0	2 (2.2)	2 (3.8)
Cognitive deficit	0	0	1 (1.1)	3 (5.8)
Myocardial infarction	0	0	2 (2.2)	2 (3.8)
Dementia	0	0	0	3 (5.8)
Burns	0	0	3 (3.3)	0
Hemiplegia	0	0	1 (1.1)	1 (1.9)
Connective tissue disorder	0	0	1 (1.1)	1 (1.9)
Stroke	0	0	0	2 (3.8)
Malignancy	0	0	0	1 (1.9)
Peptic ulcer	0	0	1 (1.1)	0
Disease syndromes associated with current infection*, n (%)				
Pneumonia	0	0	2 (2.2)	4 (7.6)
Meningitis	0	0	0	2 (3.8)
Cellulitis	6 (85.7)	10 (90.9)	82 (91.1)	44 (83.0)
Osteomyelitis	0	0	6 (6.7)	11 (20.8)
Sepsis	4 (57.1)	1 (9.1)	18 (20.0)	18 (34.0)
Arthritis	0	1 (9.1)	2 (2.2)	1 (1.9)
Endometritis†	0	0	1 (3.2)	0
Otitis media	0	0	1 (1.1)	0
Streptococcal toxic shock syndrome	0	0	4 (4.4)	4 (7.8)
Necrotizing soft tissue infection	0	0	10 (11.1)	4 (7.8)
Necrotizing fasciitis	0	0	8 (8.9)	3 (5.7)
Recent (past 14 days) triggers* n (%)				
Surgery or skin incision	0	0	3 (3.3)	2 (3.8)
Delivery†	0	0	1 (3.2)	0
Post-partum (<30 days postdelivery)†	0	0	1 (1.7)	0
Penetrating trauma	0	5 (45.5)	38 (42.2)	8 (15.1)
Blunt force trauma	1 (14.3)	1 (9.1)	15 (16.7)	6 (11.3)
Surgical wound (post-operative)	0	0	2 (2.2)	1 (1.9)

Characteristics	Age group 0–4 years (n = 7)	5–17 years (n = 11)	18–49 years (n = 90)	>50 years (n = 53)
Burns	0	0	2 (2.2)	1 (1.9)
None	6 (85.7)	6 (54.6)	35 (38.9)	37 (69.8)
Unknown	0	0	2 (2.2)	2 (3.8)

*Multiple syndromes per case allowed
†Among women only (n = 58)

Appendix Table 2. Phenotypic antimicrobial resistance testing of invasive GAS isolates, 2017-2019

Drug name	Number tested	Number with resistance*	% with resistance
Ampicillin	32	0	0
Cefepime	28	0	0
Cefotaxime	31	0	0
Ceftriaxone	32	0	0
Clindamycin	32	3	9.4
Levofloxacin	32	0	0
Penicillin	29	0	0
Tetracycline	32	6	18.8
Vancomycin	32	0	0

*Resistance testing performed at the clinical laboratories for isolates identified from blood

Appendix Table 3. Characteristics of group A *Streptococcus* isolates undergoing whole genome sequencing, 2016-2019

Characteristics	2016 clinical isolates (n=19)*	2017-2018 pharyngitis isolates (n=135)†	2017-2019 severe and invasive isolates Overall (n=127)‡	Year 1§ (n=58)	Year 2§ (n=69)
Age (years), n (%)					
0–4	6 (31.6)	2 (1.5)	6 (4.7)	4 (6.9)	2 (2.9)
5–17	5 (26.3)	66 (48.9)	8 (6.3)	3 (5.2)	5 (7.3)
18–49	7 (36.8)	49 (36.3)	68 (53.5)	27 (46.6)	41 (59.4)
>50	1 (5.3)	18 (13.3)	45 (35.4)	24 (41.4)	21 (30.4)
Female, n (%)	5 (26.3)	NS	47 (37.0)	30 (51.7)	17 (24.6)
Clinical disease, n (%)					
Invasive	2 (10.5)	0	37 (29.1)	19 (32.8)	18 (26.1)
Severe wound	5 (26.3)	0	90 (70.9)	39 (67.2)	51 (73.9)
Nonsevere wound	12 (63.2)	0	0	0	0
Pharyngitis	0	135 (100.0)	0	0	0

*Clinical GAS isolates were collected in 2016 from a convenience sample of patients with a swab or biological sample sent for culture.
†GAS pharyngitis isolates were systematically collected from 2017-2018 and defined as GAS isolated from a throat swab collected from patients with a clinical presentation consistent with pharyngitis; 12 isolates were excluded due to contamination and 2 isolates were not sequenced (NS).
‡Severe and invasive GAS isolates were systematically collected from 2017 to 2019; Invasive GAS was defined as GAS isolated from a sterile body site or from a wound with a diagnosis of streptococcal toxic shock syndrome or necrotizing soft tissue infection, including necrotizing fasciitis; Severe GAS was defined as GAS isolated from a non-sterile site and requiring hospitalization; 22 cases were identified retrospectively from the private lab with no isolate collected. An additional 8 isolates were excluded due to contamination, 1 was excluded due to high GC content, 1 was excluded due to low DOC, and 2 were not sequenced.
§Year 1 defined as March 1, 2017 to February 28, 2018 and Year 2 defined as March 1, 2018 to February 28, 2019

Appendix Table 4. Distribution of *emm* types by sample type

Emm cluster and type	2016 clinical isolates (n=19)	2017-2018 pharyngitis isolates (n=135)	2017-2019 severe and invasive isolates Overall (n=127)	Year 1* (n=58)	Year 2 (n=69)
A-C3	0	2 (1.5)	1 (0.8)	0	1 (1.5)
1	0	2 (1.5)	1 (0.8)	0	1 (1.5)
A-C4	0	3 (2.2)	0	0	0
12	0	3 (2.2)	0	0	0
D4	2 (10.5)	6 (4.4)	36 (28.3)	5 (8.6)	31 (44.9)
43	0	0	1 (0.8)	0	1 (1.5)
83	2 (10.5)	4 (3.0)	10 (7.9)	5 (8.6)	5 (7.3)
91	0	2 (1.5)	25 (19.7)	0	25 (36.2)
E1	0	25 (18.5)	8 (6.3)	7 (12.1)	1 (1.5)
4	0	3 (2.2)	0	0	0
60	0	22 (16.3)	8 (6.3)	7 (12.1)	1 (1.5)
E2	0	2 (1.5)	0	0	0
92	0	2 (1.5)	0	0	0
E3	4 (21.1)	50 (37.0)	67 (5.3)	38 (65.5)	29 (42.0)

Emm cluster and type	2016 clinical isolates (n=19)	2017-2018 pharyngitis isolates (n=135)	2017-2019 severe and invasive isolates Overall (n=127)	Year 1* (n=58)	Year 2 (n=69)
49	0	0	13 (10.2)	0	13 (18.8)
58	1 (5.3)	0	1 (0.8)	0	1 (1.5)
82	3 (15.8)	49 (36.3)	53 (41.7)	38 (65.5)	15 (21.7)
87	0	1 (0.7)	0	0	0
E4	0	5 (3.7)	1 (0.8)	0	1 (1.5)
2	0	1 (0.7)	0	0	0
28	0	2 (1.5)	0	0	0
89	0	2 (1.5)	0	0	0
114	0	0	1 (0.8)	0	1 (1.5)
E6	13 (68.4)	12 (8.9)	9 (7.1)	4 (6.9)	5 (7.2)
59	13 (68.4)	3 (2.2)	7 (5.5)	4 (6.9)	3 (4.4)
81	0	9 (6.7)	2 (1.6)	0	2 (2.9)
Clade X	0	0	2 (1.6)	2 (3.5)	0
164	0	0	2 (1.6)	2 (3.5)	0
Clade Y	0	30 (22.2)	3 (2.4)	2 (3.5)	1 (1.5)
6	0	30 (22.2)	3 (2.4)	2 (3.5)	1 (1.5)

*Year 1 defined as March 1, 2017 to February 28, 2018 and Year 2 defined as March 1, 2018 to February 28, 2019

Appendix Table 5. Distribution of e*mm* type, sequence type and antimicrobial resistance genes among the 2016 clinical isolates

emm type cluster & [sequence type]	N	Antimicrobial resistance genes: tetM n (%)	aph3 n (%)	sat4A n (%)	None detected n (%)
D4					
83 [103]	2	2 (100.0)	2 (100.0)	2 (100.0)	0
E3					
58 [176]	1	0	0	0	1 (100.0)
82 [334]	3	0	0	0	3 (100.0)
E6					
59 [172]	13	0	0	0	13 (100.0)
Overall	19	2 (10.5)	2 (10.5)	2 (10.5)	17 (89.5)

Appendix Table 6. Distribution of e*mm* type, sequence type and antimicrobial resistance genes among the 2017-2018 pharyngitis isolates

emm cluster & type / sequence type	N	Antimicrobial resistance genes: tetM	aph3	sat4/sat4A	ant6	ermC	None detected
A-C3							
1 [28]	2	0	0	0	0	0	2 (100.0)
A-C4							
12 [36]	3	0	0	0	0	0	3 (100.0)
D4							
83 [103]	4	4 (100.0)	4 (100.0)	4 (100.0)[a]	0	0	0
91 [12]	2	0	0	0	0	0	2 (100.0)
E1							
4 [39]	3	0	0	0	0	0	3 (100.0)
60 [53]	22	22 (100.0)	0	0	0	0	0
E2							
92 [82]	2	2 (100.0)	2 (100.0)	2 (100.0)[a]	2 (100.0)	2 (100.0)	0
E3							
82 [334]	49	0	0	0	0	0	49 (100.0)
87 [62]	1	0	0	0	0	0	1 (100.0)
E4							
2 [55]	1	0	0	0	0	0	1 (100.0)
28 [458]	2	0	0	0	0	0	2 (100.0)
89 [101]	2	0	0	0	0	0	2 (100.0)
E6							
59 [172]	3	0	0	0	0	0	3 (100.0)
81 [837]	9	0	0	0	0	0	9 (100.0)
Clade Y							
6 [382]	30	0	0	0	0	0	30 (100.0)
Overall	135	28 (20.7)	6 (4.44)	6 (44.4)	2 (1.5)	2 (1.5)	107 (79.3)

[a] The 4 *emm* type 83 isolates had sat4A detected and the 2 *emm* type 92 isolates had sat4 detected

Appendix Table 7. Distribution of e*mm* type, sequence type and antimicrobial resistance genes among the 2017-2019 severe and invasive isolates

***emm* cluster & type / sequence type**	**N**	**Antimicrobial resistance genes**			
		**tetM**	**aph3**	**sat4A**	**None detected**
A-C3					
1 [28]	1	0	0	0	1 (100.0)
D4					
43 [3]	1	0	0	0	1 (100.0)
83 [103]	10	9 (90.0)	9 (90.0)	9 (90.0)	1 (10.0)
91 [12]	25	0	0	0	25 (100.0)
E1					
60 [53]	8	8 (100.0)	0	0	0
E3					
49 [433]	13	0	0	0	13 (100.0)
58 [176]	1	0	0	0	1 (100.0)
82 [334]	53	0	0	0	53 (100.0)
E4					
114 [188]	1	1 (100.0)	1 (100.0)	1 (100.0)	1 (100.0)
E6					
59 [172]	7	0	0	0	7 (100.0)
81 [837]	2	0	0	0	2 (100.0)
Clade X					
164 [53]	2	2 (100.0)	0	0	0
Clade Y					
6 [382]	3	0	0	0	3 (100.0)
Overall	127	20 (15.8)	10 (7.9)	10 (7.9)	107 (84.3)

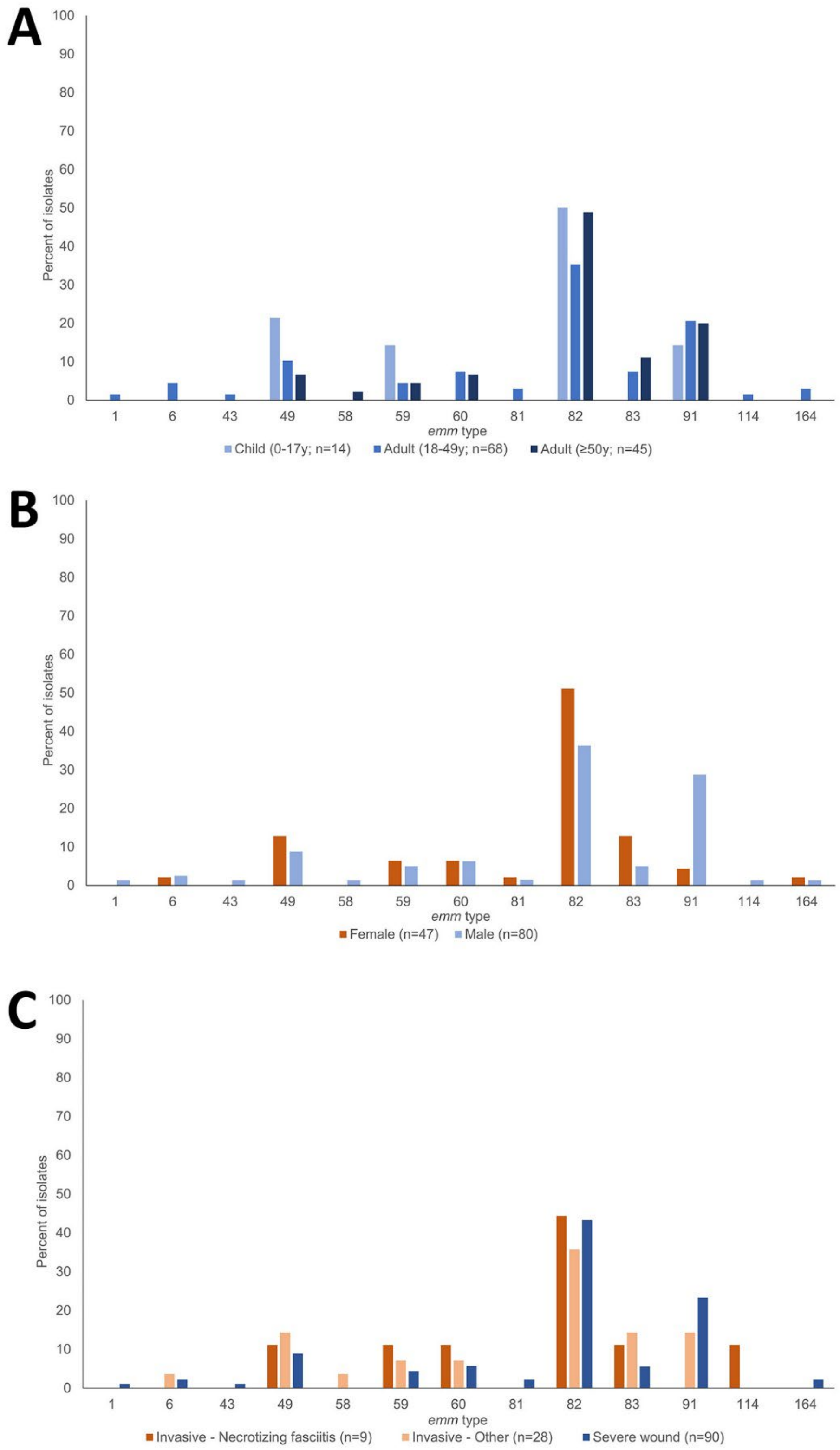

Appendix Figure 1. Distribution of *emm* types among invasive and severe group A *Streptococcus* cases by A) age, B) sex, and C) clinical syndrome

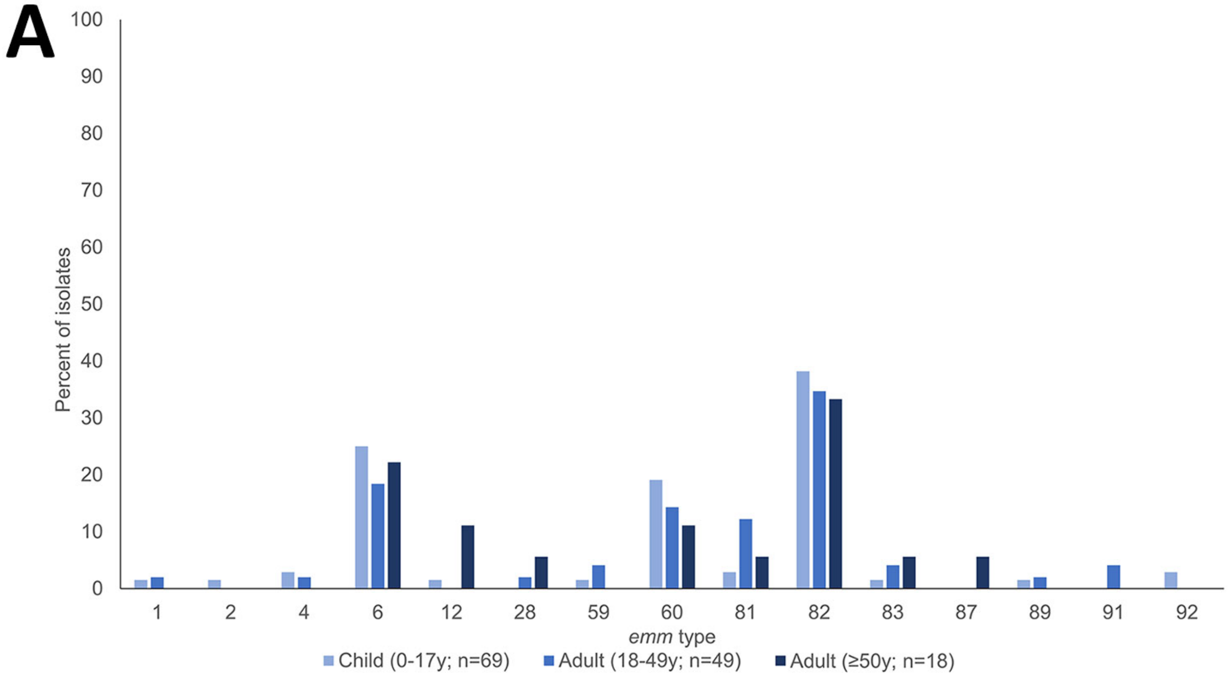

Appendix Figure 2. Distribution of *emm* types by A) age and B) year among pharyngitis cases
